# Contributions of Age-Related Thymic Involution to Immunosenescence and Inflammaging

**DOI:** 10.1186/s12979-020-0173-8

**Published:** 2020-01-20

**Authors:** Rachel Thomas, Weikan Wang, Dong-Ming Su

**Affiliations:** 1 Cell Biology, Immunology, and Microbiology Graduate Program, Graduate School of Biomedical Sciences, Fort Worth, Texas 76107 USA; 2 Department of Microbiology, Immunology, and Genetics, University of North Texas Health Science Center, Fort Worth, Texas 76107 USA

**Keywords:** Thymic aging, Age-related thymic involution, Central tolerance, Negative selection and regulatory T (Treg) cell generation, Immunosenescence and inflammaging, Rejuvenation

## Abstract

Immune system aging is characterized by the paradox of immunosenescence (insufficiency) and inflammaging (over-reaction), which incorporate two sides of the same coin, resulting in immune disorder. Immunosenescence refers to disruption in the structural architecture of immune organs and dysfunction in immune responses, resulting from both aged innate and adaptive immunity. Inflammaging, described as a chronic, sterile, systemic inflammatory condition associated with advanced age, is mainly attributed to somatic cellular senescence-associated secretory phenotype (SASP) and age-related autoimmune predisposition. However, the inability to reduce senescent somatic cells (SSCs), because of immunosenescence, exacerbates inflammaging. Age-related adaptive immune system deviations, particularly altered T cell function, are derived from age-related thymic atrophy or involution, a hallmark of thymic aging. Recently, there have been major developments in understanding how age-related thymic involution contributes to inflammaging and immunosenescence at the cellular and molecular levels, including genetic and epigenetic regulation, as well as developments of many potential rejuvenation strategies. Herein, we discuss the research progress uncovering how age-related thymic involution contributes to immunosenescence and inflammaging, as well as their intersection. We also describe how T cell adaptive immunity mediates inflammaging and plays a crucial role in the progression of age-related neurological and cardiovascular diseases, as well as cancer. We then briefly outline the underlying cellular and molecular mechanisms of age-related thymic involution, and finally summarize potential rejuvenation strategies to restore aged thymic function.

## Introduction

**Fig. 1:**
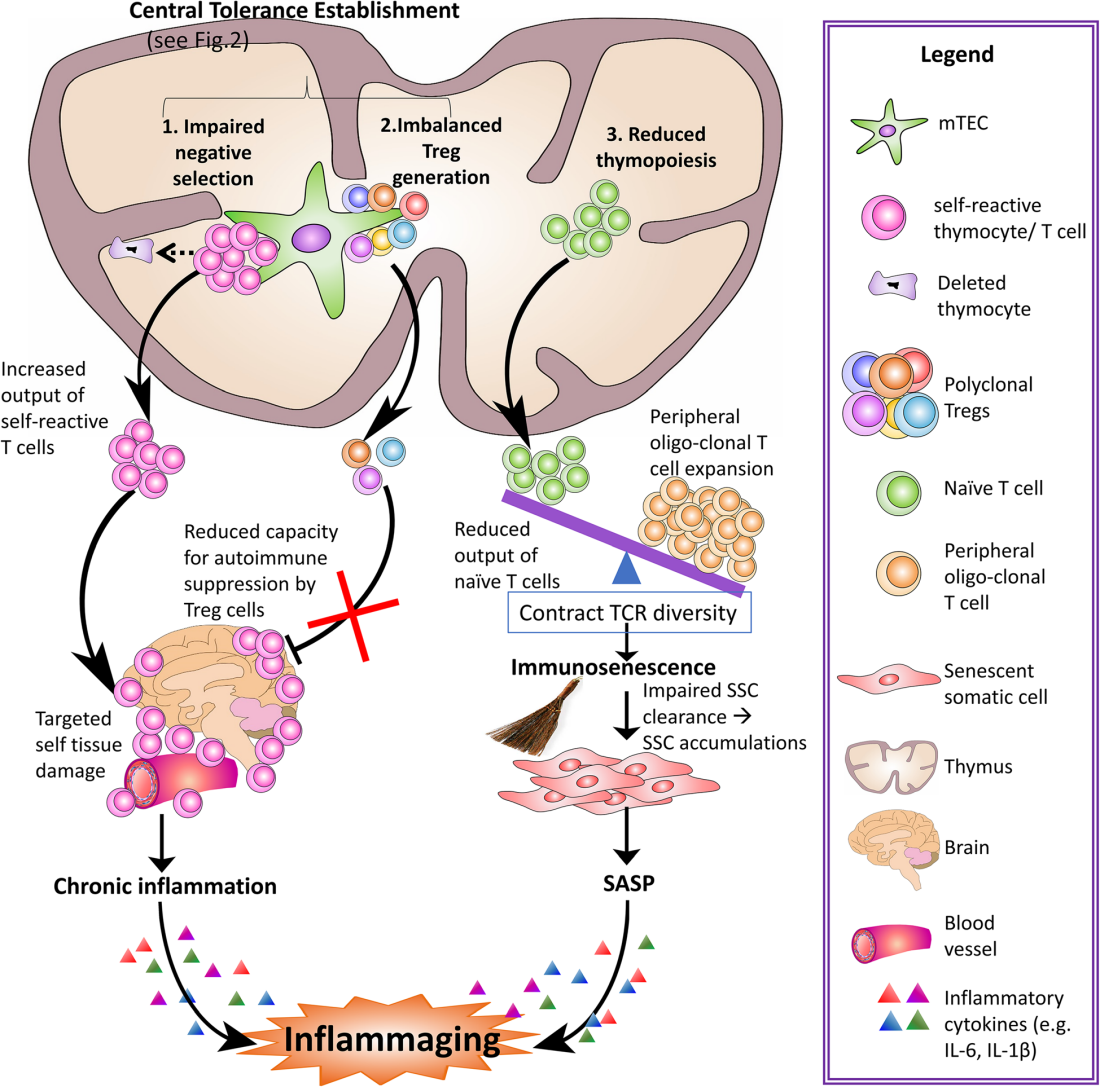
Intersection of immunosenescence and inflammaging is associated with age-related thymic involution. The aged, involuted thymus exhibits ineffective central tolerance and declined thymopoiesis. The ineffective central tolerance includes (1) impaired negative selection, which leads to the increased output of self-reactive T cells that attack self-tissues/organs, and (2) imbalanced generation of tTreg TCR repertoire, which fails to sufficiently suppress self-reactive T cell–mediated autoimmune responses. Autoimmune responses lead to tissue damage and thus cause chronic inflammation, which is one of the contributors to inflammaging. Reduced thymopoiesis leads to decreased output of naïve T cells for the clearance of senescent somatic cells (SSCs) and the expansion of oligo-clonal T cells in the aged periphery lack sufficient clearance capacity, which allows for SSC accumulation. SSCs are an important source of SASP, another contributor to inflammaging

The aged immune system has various characteristics. One of which is immunosenescence, which describes the vast and varied changes in the structure and function of the immune system as a result of age [1–4]. Many of the early observations, such as reduced ability to fight new infections, diminished vaccine immunity [5], and reduced tumor clearance [6, 7] are generally categorized as immune insufficiencies. Immunosenescence is not due to the lack of immune cells, but due to reduced immune repertoire diversity, attributed to insufficient production of naïve immune cells and amplified oligo-clonal expansion of memory immune cells. Immunosenescence is therefore linked to the thymus. Natural aging causes the thymus to progressively atrophy, a process called thymic involution. This phenomenon is readily observed in most vertebrates [8] and results in structural alterations, as well as functional decline, ultimately resulting in significantly decreased thymic output of naïve T cells [9–11] that reduces the diversity of the T cell antigen receptor (TCR) repertoire, culminating in disrupted T cell homeostasis (Fig. 1, #3 right side).

The second characteristic of aged immunity is termed inflammaging. Inflammaging describes the elevated self-reactivity in the elderly, resulting in the typical chronic, low-grade, but above baseline, systemic inflammatory phenotype observed in the absence of acute infection [12–19]. Inflammaging was originally attributed to somatic cell senescence-associated secretory phenotype (SASP) [20–22] and chronic innate immune activation. In recent years, however, the contribution of aged adaptive immune components and specifically self-reactive T lymphocytes, has been realized [13, 23], as a probable primary contributor to the age-related development of subclinical autoimmune predisposition. Although immunosenescence and inflammaging appear to be opposing phenotypes, they comprise two sides of the same coin [24] when attempting to holistically understand age-related immune dysfunction [3, 4, 24, 25]. It has been proposed that the basal inflammatory state in the elderly, defined by inflammaging, greatly contributes to many age-related degenerative diseases [23], including metabolic diseases, such as Type-II Diabetes (as a complication of pancreatitis), neurodegenerative diseases, such as Alzheimer’s disease, and cardiovascular diseases, such as atherosclerosis [18, 23, 26–28].

T lymphocyte (T cell) development and selection occurs in the thymus [29]. Included in this process is central tolerance establishment (Fig. 1, #1 and #2 top left), which occurs via two mechanisms. First is thymocyte negative selection, during which the majority of self (auto)-reactive T cells are depleted from the repertoire via apoptosis [30]. Second is the generation of CD4 single positive (CD4^SP^) FoxP3^+^ regulatory T (Treg) cells [31], whose primary function is to suppress T cell-mediated self-reactivity and preserve immune homeostasis in the periphery [32]. These arms of central T cell tolerance work in tandem, and Treg cells most likely compensate for imperfections of negative selection, as some self-reactive T cells escape negative selection [33]. With age, however, the atrophied thymus declines in its capacity to establish central tolerance, thereby, causing increased self-reactive T cells to escape to the periphery and participate in the process of inflammaging.

Historically, there have been two schools of thought regarding theoretical causes of age-related decreased thymopoiesis. First is the idea of defective hematopoietic stem cells, since there are reduced numbers of hematopoietic stem cell (HSC) progenitors produced by aged bone marrow (BM) [34]. It therefore follows that there are fewer early T-cell progenitors (ETP) entering the thymus from the BM, resulting in shrinkage of the thymus [35]. Second is the idea of a defect in stromal niches of the BM [36, 37] or thymus [38, 39]. Therefore, age-related hallmarks of thymic involution primarily occur within the thymic niche and then extend to impact the development of ETPs.

It is our belief that the latter theory is more substantiated in light of recent advances, and that the extensive age-related alterations in thymic structure and microenvironment contribute most to the diminished thymopoiesis observed in the elderly [11, 38]. Thymic epithelial cells (TECs) are the primary thymic stromal cells and include two sub-populations: medullary TECs (mTECs) and cortical TECs (cTECs). These two cell populations are distinct in their thymic localization, functions during thymocyte development, and molecular expression patterns [40, 41]. Since the advent of cell-type specific conditional gene knock-out (cKO) models, compelling evidence show that age-related thymic atrophy is tightly associated with postnatal TEC homeostasis, regulated by TEC autonomous transcription factors (TFs), such as Forkhead box N1 (*FOXN1*) [42]. To this end, rejuvenation of age-related thymic involution by developing *FOXN1*-TEC axis-based therapeutics is reasonable, although other strategies are under investigation [43].

In this review, we will discuss recent research progress exploring how age-related thymic involution contributes to inflammaging progression in conjunction with immune insufficiency, resulting in reduced clearance of the senescent somatic cells (SSCs), coupled with increased T cell-mediated self-reactivity and inflammation. We will outline the differences in general senescence and immunosenescence as it pertains to inflammaging and age-related immune dysregulation. We will describe how the involvement of T cell adaptive immunity in mediating inflammaging plays a crucial role in the progression of age-related neurological and cardiovascular diseases, as well as cancer. Finally, we will briefly outline the underlying cellular and molecular mechanisms of age-related thymic involution and summarize potential rejuvenation strategies to restore aged thymic function. Finding new ways to attenuate the impacts of age-related thymic involution on inflammaging and immunosenescence is of great clinical significance in an era refocusing medicine toward healthy aging.

## Contributions of Thymic Involution to T Cell Immune System Aging

Since T cell immune system aging mainly includes two aspects: immunosenescence and inflammaging, in this section we discuss recently published papers about how they intersect, how they are induced, and how age-related thymic involution participates in these processes. We outline this intricate relationship between immunosenescence and inflammaging associated with age-related thymic involution in Fig. 1.

### Intersection of Immunosenescence and Inflammaging

When discussing hallmarks of biological aging, seven overarching pillars [44] are thought to collapse, namely: decreased adaptation to stress, loss of proteostasis, exhaustion of stem cells, derangement of metabolism, macromolecular damage, epigenetic dysregulation, and intercellular communication disorder. These changes are intricately linked through the crossroads of immunosenescence and inflammaging [23, 45], which characterize immunology of aging.

Conventional senescence is a general term usually denoting somatic cellular senescence, referring to permanent or durable cell-cycle arrest first observed in cultured fibroblasts. The original observations leading to the discovery of senescence were not fully acknowledged by the scientific community because the initial observations were described in *in vitro* cultured cells, although this group believed there to be cell intrinsic factors leading to the observed “degeneration” of the cells [46]. It was later demonstrated that senescence occurs *in vivo* and has since been more adequately defined as cells exhibiting permanent cell cycle arrest, lack of proliferation, expression of corresponding anti-proliferation markers, such as p16^INK4a^ and senescence-associated β galactosidase (SA-β-gal), shortened telomeres, and activation of DNA-damage signaling cascades. The characteristics of somatic cell senescence have recently been significantly reviewed elsewhere [47, 48].

Somatic cellular senescence is believed to be advantageous as an evolutionary protection against cancer development [47]. However, senescence of somatic cells during aging is thought to significantly contribute not only to degeneration of aged tissue function if SSCs are accumulated in certain organs, but also to the systemic inflammatory milieu via induction of SASP [18–23, 49]. This largely pro-inflammatory cellular secretion pattern induces increased basal levels of serum IL-6 and IL-1, as well as matrix metalloproteinases (MMPs) [18, 47]. SASP has therefore been cited as a major contributor to inflammaging [18, 19, 23, 49]. Some of the mechanisms suggested to trigger cellular senescence are prolonged or chronic insults that accumulate over time, such as oxidative stress, gradual telomere shortening, and chronic infections. One additional characteristic of senescent cells is that they actively resist apoptosis [47]. The anti-apoptotic pathways involve many factors including downregulation of Capsase-3 and increased Cyclin-dependent kinase inhibitors, p16 and p21 [50]. More recently, histone modification studies have implicated altered expression ratios of Bcl-2 and Bax family genes in mediating the anti-apoptotic phenotype of senescent fibroblasts [51].

Immunosenescence is a much broader term that encompasses all age-related changes to the immune system, both innate and adaptive [27, 52]. The primary hallmarks of immunosenescence are dampened immune responses to new infection or vaccination, and diminished anti-tumor immunosurveillance, including altered immune response phenotypes in activated T cells, increased memory T cell accumulation, and an inverted T lymphocyte subset ratio [52]. Immunosenescence in T cells [53] is commonly termed “cellular exhaustion”. This is usually characterized as loss of co-stimulatory surface molecule CD28 and expression of Tim-3, in addition to the other features of cellular senescence [54]. T cell exhaustion differs from conventional senescence because of upregulation of surface markers such as PD-1 and Tim-3. Additionally, this type of growth arrest is not permanent, as blocking PD-1 can reverse T cell exhaustion, as demonstrated by recent clinical trials [54, 55]. This unique type of growth arrest in T cells is primarily due to prolonged or chronic TCR/antigen stimulation.

Recently, a link between immunosenescence and somatic cellular senescence has been established [56, 57], in which the SSCs are no longer homeostatically reduced by the immune response. This results when natural killer (NK) cells, macrophages, astrocytes, and T cells undergo diminished chemotaxis toward accumulated SSCs for targeted depletion [56–58]. The mechanisms by which T cells deplete accumulated SSCs could include CD8^+^ cytotoxic T lymphocytes (CTLs), CD4^+^ Th1-like cells producing cytotoxic inflammatory cytokines (such as IFN-γ), and Th2-like cells producing IL-4 and TGF-β [56, 57]. In addition to diminished chemotaxis, there is also dampened phagocytosis by neutrophils and macrophages associated with age that facilitates SSC accumulation [59, 60]. This ultimately results in increased production of SASP [21], which significantly contributes to inflammaging and subsequent development of age-related diseases [22, 61]. This intersection of inflammaging and immunosenescence with age-related diseases remains unclear, but many groups are currently exploring various models to further elucidate the impact of inflammaging and immunosenescence on age-related disease progression [23, 62].

### Underlying Etiology of Immunosenescence and Inflammaging

There are several proposed components underlying immunosenescence and inflammaging etiology. In addition to cellular SASP secretions which contribute to inflammaging as discussed above, chronic innate immune activation due to long-term latent or persistent viral infection, for example, with members of the *Herpesviridae* family, have been proposed to contribute to low level pro-inflammatory cytokine production [17]. Most notably, cytomegalovirus (CMV), infection has been explored as a potential biomarker in aging human patients [17, 63–65]. For example, several longitudinal studies of aging adults saw correlations with CMV sero-positivity and increased morbidity [66, 67]. Importantly, the role of the aged adaptive immune responses to self-tissues (in the absence of acute infection), primarily induced by the T cell compartment, has been found to be a major player in the onset and progression of inflammaging [12, 13] and associated with immunosenscence [14, 68]. The aged, atrophied thymus, continues to select T cells throughout the lifetime of the individual. However, the atrophied thymus is less able to negatively select self-reactive T cells, releasing these harmful, self-reactive T cells to the periphery, thereby, increasing subclinical autoimmune predisposition in the elderly [14]. Additionally, age-related thymic atrophy results in reduced output of functional naïve T cells, or recent thymic emigrant cells (RTEs) [9], over time [69]. Since peripheral T cell numbers remain unchanged or relatively elevated in aged individuals [70–72], the reduced thymic output in combination with peripheral oligo-clonal expansion of memory T cells, which occupy immunological space in the periphery [73–75], results in an overall contracted TCR repertoire diversity [9, 76–78] thereby inducing immune insufficiency (immunosenescence).

### Thymic involution directs Immunosenescence and Inflammaging

Given both the altered output of naïve T cells and disruption of central tolerance establishment, it follows that thymic involution contributes to T cell-associated immunosenescence and inflammaging. Herein, we review recently determined evidence in this field.

As indicated above, an age-related subclinical autoimmune predisposition induced by adaptive immune reaction to self-tissues by self-reactive T cells has recently been recognized as a potential factor underlying inflammaging [13, 23]. This results mainly from that increased output of self-reactive T cells by the atrophied thymus, which should be depleted through negative selection as the first boundary for preventing self-reactivity. Treg cells suppress self-reactivity as the second frontier to prevent self-inflicted tissue damage. However, aged Treg cells usually are unable to do so [79], potentially due to the lack of Treg TCR diversity, as seen in an autoimmune diabetes model [80, 81]. These changes are attributed to defects in central tolerance establishment during the thymocyte development process, encompassing negative selection and thymic Treg (tTreg) cell generation.

#### Defective Negative Selection

**Fig. 2:**
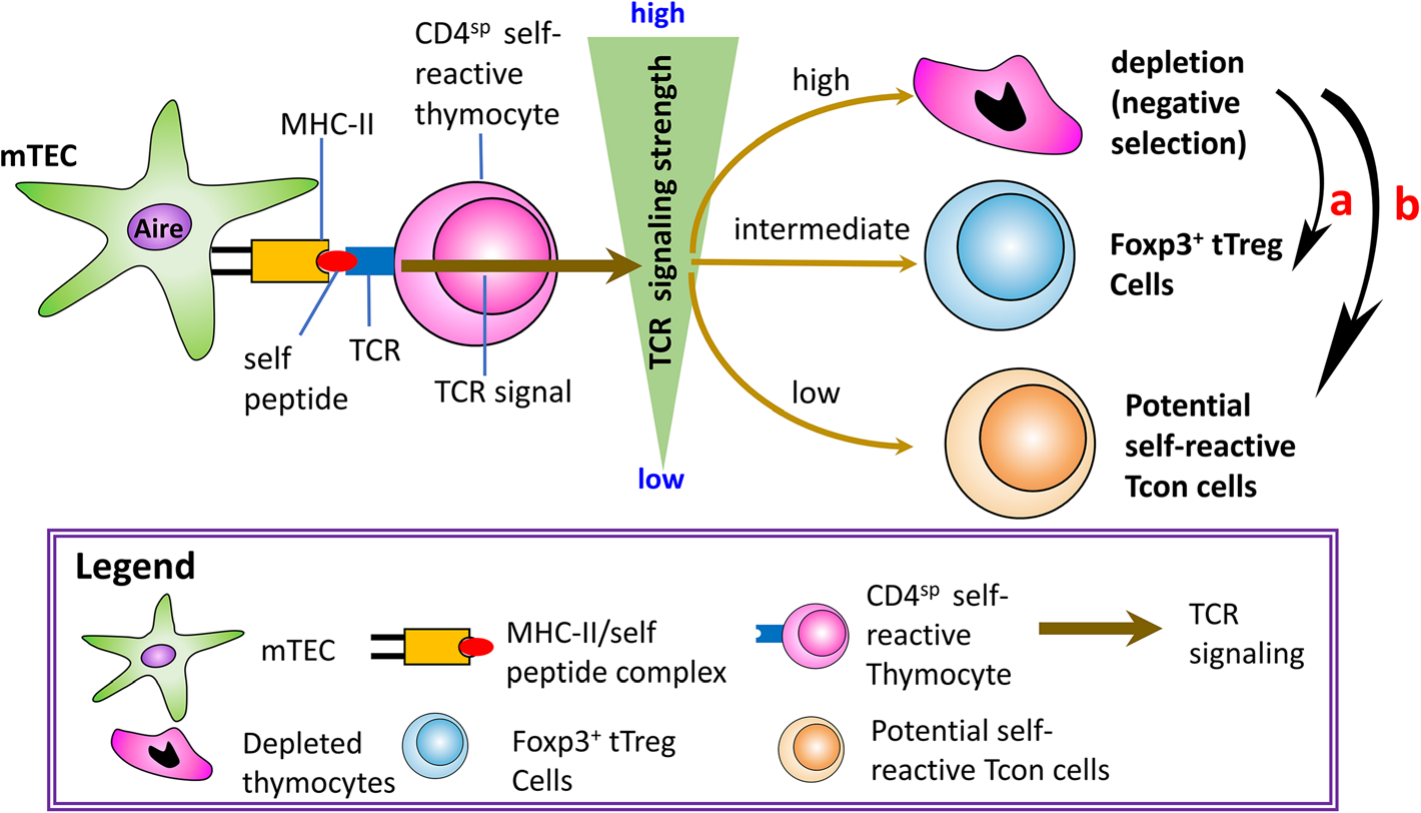
TCR signaling strength decides self-reactive CD4^sp^ T clone fates. Interaction between MHC-II/self peptide complex on mTEC and self-reactive TCR on CD4^sp^ thymocyte produces three types of signaling strength: (1) a strong signal leads to negative selection, resulting in thymocyte depletion; (2) an intermediate signal leads to tTreg generation; (3) a weak signal results in thymocyte differentiation into T conventional (Tcon) cells. We hypothesize that age-related thymic involution shifts signaling strength from strong to intermediate and relatively enhances polyclonal tTreg generation (black arrow—a) ; while in some cases, antigen-specific interactions exhibit an even weaker signal, resulting in diminished antigen-specific tTreg cells and increased antigen-specific Tcon cells (black arrow—b)

Under the current paradigm, negative selection is the systematic removal of thymocytes expressing a TCR that exhibits high affinity for self-peptides presented by major histocompatibility complex class II (MHC-II) on mTECs [30, 41, 82]. In support of this paradigm, it has been shown that when these high affinity TCRs receive strong signaling, negative selection follows via apoptosis of the thymocyte [83, 84]. However, overall TCR signaling strength is a culmination of TCR affinity for the self-peptide and avidity, or the combination the of affinity of TCR for self-peptide/MHC-II (self-pMHC-II) complexes and the number of TCR/self-pMHC-II interactions that occur (Fig. 2). Therefore, if thymocyte-dependent factors (i.e. TCR affinity and number) of self-reactive thymocytes are unchanged, then TCR signaling strength depends on the efficiency of self-pMHC-II expression by mTECs. Since aging induces mTEC defects, such as decreased expression of autoimmune regulator (*AIRE*) and MHC-II, there is reduced capacity for self-pMHC-II ligand expression [85, 86]. Therefore, we suggest that a strong signaling strength shifts either to an intermediate strength, which favors CD4^SP^FoxP3^+^ tTreg cell generation (Fig. 2, arrow-a), or to an even lower (weak) strength, resulting in the release of self-reactive thymocytes (Fig. 2, arrow-b) with the potential to initiate self-reactivity and auto-inflammation.

The *FOXN1* cKO mouse model has proven to be a beneficial model for studying the capacity for efficient self-pMHC-II ligand expression, because it maintains young hematopoietic precursor cells and a young periphery in order to isolate the effects of TEC defects associated with thymic involution. We showed that thymic involution disrupts negative selection, as revealed by the enhanced release of self-reactive T cells recognizing interphotoreceptor retinoid-binding protein (IRBP) from the atrophied thymus of *FOXN1* cKO mice compared to control [13]. This result was confirmed using a mock self-antigen model in which expression of ovalbumin (under control of the *AIRE*-regulated rat insulin promoter) was decreased in the involuted thymus compared to control [85].

#### Thymic-Derived Regulatory T Cell Generation

The second arm of central tolerance induction is the generation of tTreg cells, which function to suppress autoimmune or chronic immune reactions in the periphery as peripheral Treg (pTreg) cells. It is proposed that Treg cells compensate for imperfections in negative selection [33] that allow some self-reactive T cells to enter the periphery. It is currently accepted that 80 - 95% of pTreg cells are directly generated in the thymus, as opposed to Treg cells that are induced in the periphery [87–89]. Under the current paradigm, the processes of both negative selection and tTreg generation in the thymus utilize the same set of agonist self-peptides [87, 90]. In this setting, TCR signaling strength determines whether developing thymocytes are deleted via negative selection or enter the tTreg cell lineage. As described in the previous section, TCR signaling strength is cumulative of TCR affinity and avidity, all other thymic microenvironmental variables, such as IL-2, being equal. Moreover, strong signaling induces apoptosis of highly self-reactive thymocytes, while intermediately high signaling leads to tTreg generation (Fig. 2, arrow-a). Weak signaling results in the survival of thymocytes that differentiate into conventional T (Tcon) cells (Schematic diagram in Fig. 2, arrow-b) [41, 87].

As mentioned earlier, the mTECs of the aged, involuted thymus are less apt to express self-antigens, and this could definitively dampen the overall avidity of TCR signaling received by developing thymocytes. We have observed a relatively increased tTreg generation by the atrophied thymus, which showed no change in overall tTreg numbers, but an increased %tTreg:%tTcon cell ratio in the aged, atrophied thymus compared to controls with the normal thymus [85]. This phenotype was also observed in a mouse model with partial defects in MHC-II through microRNA inhibition [91]. We believe this to be a demonstration of the atrophied thymus attempting to compensate for defective negative selection [13] in order to maintain central T cell tolerance in the elderly.

Additionally, several studies investigating the effects of diminished ability for thymic self-antigen presentation in mTECs, such as *AIRE* gene knock, have shown similar results of no change in overall Treg production. In combination with age-related accumulation of pTreg cells in the periphery of mice and humans [73–75, 92], the relative proportion of pTreg cells is increased in the elderly [93], though these aged pTregs exhibit unimpaired functionality [94–96]. Therefore, why is the increased or unchanged proportion of Treg cells unable to successfully manage self-reactivity in the elderly? We hypothesize that despite increased polyclonal Treg cells, certain self-tissue-specific Treg cells are reduced or imbalanced with self-reactive T effector (Teff) cells due to thymic atrophy, creating holes in the Treg repertoire. There are several mouse models using *AIRE* gene alterations that result in similar defects in self-antigen presentation seen in the aged thymus that support our hypothesis.

One study assessed the effects of *AIRE* knock-out thymus on generation of a particular self-antigen-specific tTreg population, namely for the TCAF3 epitope of a prostate antigen, and saw a significant redirection of this TCR-bearing thymocyte from the tTreg to the Tcon lineage [97] (Fig. 2, arrow-b indicated). These redirected TCAF3-specific Tcon cells were able to infiltrate the prostate tissue and cause visible lesions, but few TCAF3-specific Treg cells were observed [97, 98].

Other studies investigating defects in mTEC self-peptide expression due to specific self-protein knock-out [99] are beginning to indicate that similar impairments exhibited by the aged thymus may negatively impact antigen-specific (monoclonal) tTreg generation despite an unchanged or increased total (polyclonal) tTreg population. In contrast, these age-related thymic impairments seem to increase output of Tcon cells recognizing the same self-antigens and may contribute to increased peripheral self-tissue damage and inflammation [100]. Further investigation will hopefully shed more light on how these subtle deficits in central tolerance establishment by the aged thymus impact the aged Treg TCR repertoire, in spite of a relatively increased aged polyclonal Treg population [85].

## Participation of Immunosenescence and Inflammaging in Age-Related Diseases

Immunosenescence and inflammaging begin as largely subclinical conditions, which eventually underlie age-related diseases. High-risk diseases in the elderly including neurodegenerative diseases, cardiovascular diseases, and late-life cancers [101–106] are associated with a persistent, chronic pro-inflammatory status and impaired regulation of aberrant pro-inflammatory cells due to immunosenescence in aged individuals [56, 107, 108].

### Age-related neurodegenerative diseases

Most age-related neurodegenerative diseases, such as Alzheimer’s disease (AD), are associated with immunosenescence and/or inflammaging, which cause structural and functional disturbances of the blood-brain barriers (BBB) [109, 110], thereby, leading to the infiltration of immune cells [101, 109, 111] into the central nervous system (CNS). However, whether these disease outcomes are a cause or an effect of the imbalanced pro-inflammatory and anti-inflammatory immune cells is under investigation [112].

Among these immune cells are IFN-γ-producing Th1 cells and IL-17A-producing Th17 cells that are pro-inflammatory. They interact with the CNS resident microglia and exacerbate AD [113–115]. Treg cells in AD play dual roles, either pathogenic or protective in various animal studies [116, 117]. Conversely, Th2 cells specific for amyloid beta (Aβ), which is a peptide that is accumulated in the AD brain [118], ameliorate AD in mice, showing improved cognition and reduced burden of Aβ depositions.

In addition to the BBB, the choroid plexus (CP) is also an important barrier that maintains CNS homeostasis [111, 119, 120]. The CP harbors CD4^+^ Th1 cells producing IFN-γ that stimulate CP epithelium to express leukocyte trafficking factors and recruit small numbers of leukocytes, including T cells and monocyte-derived macrophages. In contrast to the pro-inflammatory Th1 phenotype associated with disease exacerbation when in the CNS parenchyma, the IFN-γ producing Th1 cells in the CP support CNS tissue repair and maintain normal cognitive conditions [121, 122]. However, insufficient Th1 function occurs in the aged CP, leading to reduced IFN-γ and premature cognitive impairment in several mouse models [119, 123]. This decreased Th1 immune response might represent one of the profiles of immunosenescence [124], but the definite mechanisms remain to be explored.

The role of infiltrating T cells in the CNS, including effector T (Teff) and Treg cells, is another critical element in AD, which could be associated with age-related thymic involution. Teff cells, especially CNS-infiltrating Th1 cells, are recognized as pathogenic by multiple studies [112]. For example, Th1 cells specific for Aβ-antigen in the AD brain was verified to promote the disease in mice [114]. Based on this fact, it remains an interesting question whether the altered negative selection of antigen-specific T cells in the aged thymus is more favorable for the development of Aβ-specific Teff cells, and thereby could potentially predispose aged individuals to AD.

Additionally, Treg cells could play dichotomous roles in AD, either protective or pathogenic [101] likely depending on their location in the brain. The fundamental function of Treg cells in immune response is to suppress the activity of other immune cells including Teff and myeloid leukocytes. Thus, they are generally believed to inhibit neuroinflammation inside the CNS [125]. However, Treg cells, which are residing at the CP, but not infiltrating into the CNS, can be detrimental for AD, because the CP is an important gateway for leukocyte trafficking into the CNS to maintain its homeostasis [121, 122]. However, CP leukocyte trafficking can be suppressed by Treg cell-produced IL-10 [126]. Additionally, Treg cells can directly inhibit the expression of leukocyte trafficking molecules by the CP, which was verified to aggravate AD in an animal model [117]. Treg cells are accumulated in the periphery with advancing age in both mice and humans [73–75, 92, 93], partially due to the relatively enhanced Treg generation in the aged, atrophied thymus [85], and Treg cells also have an increased suppressive function in aged mice [96]. Therefore, the negative effects of Treg cells in the CP and increased Treg proportion and function in the aged periphery could be an important aspect for AD susceptibility and provide a potential therapeutic target.

### Age-related cardiovascular diseases

Age is also a predominant risk factor for cardiovascular diseases [127, 128], the principal pathological consequences of which involves vascular endothelial dysfunction and arterial stiffness. These basic pathologies are associated with immunosenescence and inflammaging, particularly on the cardiovascular wall, and lead to hypertension, atherosclerosis, and ultimately heart failure [129, 130].

In recent years, accumulating evidence has implicated the senescent T cell immune system in the pathogenesis of cardiovascular diseases, such as atherosclerosis, which is proposed to be related to thymic involution [28], as well as links to clonal expansion of senescent T cells and accumulation of effector memory T cells in the elderly [130]. Apolipoprotein B (ApoB) is the major apolipoprotein constituent of low-density lipoprotein (LDL), which is a causal agent for atherosclerosis [131, 132]. Although T cells are not the primary pathogenic cells in atherosclerotic lesions, ApoB-100-specific T cells were reported in an atherogenic mouse model [133] and ApoB p18-specific Treg cells were found in healthy individuals [134]. This indicates that age-related thymic involution might predispose elderly individuals to atherosclerosis by biasing the balance of ApoB-specific Teff versus Treg cells.

It is known that macrophages are the primary pathogenic cells in atherosclerotic lesion onset. The accumulating CD8^+^ CD28^null^ CD27^-^ senescent T cells [135, 136] on the inflammatory cardiovascular wall constantly produce IFN-γ, which activates macrophages to release MMPs for extracellular matrix degradation [137]. This is an important underlying mechanism of atherosclerosis etiology related to T cells. In addition, CD4^+^ CD28^null^ senescent T cells are relevant to the recurrence of acute coronary events [138]. Circulating CD4^+^ effector memory T cells were reported to be associated with atherosclerosis in humans and in mouse models [139], providing further indication for the role of immunosenescence in cardiovascular disease.

### Late-Life Cancers

There is substantial knowledge regarding aged immune function and cancer. Immunosenescence with advanced age is known to decrease cancer cell immunosurveillance [140, 141], and inflammaging creates a favorable cytokine microenvironment for tumorigenesis [106, 142]. However, knowledge about how age-related thymic involution directly contributes to tumor development is insufficient.

Declined immunosurveillance of cancer cells is related to reduced thymopoiesis leading to an altered or contracted TCR repertoire diversity [143]. If the range of tumor antigen recognition is narrowed by thymic involution, the aged T cell immune system will be less apt to clear cancerous cells. Likewise, if the proportion of pro-tumorigenic TCRs is biased, the risk for cancer development is increased. For example, a pro-tumorigenic γδ-T cell subset bearing Vγ6 and Vδ1 TCR chains, which is related to a higher risk of cancer development, was reported to be accumulated in aged mice [144, 145], but it remains undetermined whether this pro-tumorigenic γδ-T cell population is increased by the altered negative selection in the aged thymus or by clonal expansion in the aged periphery.

Treg cells, on the other hand, contribute substantially to the suppression of anti-tumor T cell responses, and they frequently accumulate in the tumor microenvironment, dampening anti-tumor immunity [146, 147]. Numerous studies have shown that cancer patients have increased Treg cells in peripheral blood and tumor microenvironment [148–151]. For example, elderly lung cancer patients have more Treg cells in peripheral blood than age-matched controls [152]. This corresponds to the peripheral accumulation of Treg cells and the potentially enhanced tTreg generation by the aged thymus [85], which could be an important factor predisposing elderly individuals to late-life cancer.

An important aspect for cancer prognosis is metastatic relapse, which typically occurs several years after removal of the primary tumor and treatment with adjuvant therapy. The question is where the residual tumor cells hide during chemo- and/or radiotherapy. It has been shown that lymphoid cancers can hide in the thymus in mice [153, 154]. Also, we recently reported that in mice the atrophied thymus can be a pre-metastatic cancer reservoir to protect non-lymphoid, solid cancer cells from chemotherapy because the thymus provides an inflammatory microenvironment favorable for solid tumor cell dormancy during chemotherapy [155].

Inflammation is a double-edged sword that is necessary for anti-tumor responses [156, 157], but it can also induce drug resistance in the tumor cells [158, 159]. Particularly, chronic inflammation is associated with increased risk of cancer, as supported by many studies [160–162]. Inflammation-driven cancers are induced by inflammatory cytokines, initiating or promoting multiple processes in tumorigenesis including cellular mutations, metastasis, tumor growth, and angiogenesis [142, 163]. For example, macrophages and T cells release TNFα which can exacerbate DNA damage [164] and tumor-associated macrophages secrete macrophage migration inhibitory factor that dampens p53-dependent protection [165]. TNFα was also found to increase cancer metastasis to the lung [166] and the liver [167] in animal models. Additionally, tumor growth is promoted by IL-6 via the IL-6/JAK2/STAT3 pathway in kidney, lung and breast cancer [168], and angiogenesis in prostate cancer patients was found to be associated with TGFβ [169]. These examples demonstrate the mutagenic potential of several classic cytokines.

One additional component contributing to age-related increased cancer incidence is the skew toward myelopoesis compared to lymphopoesis that is readily observed in both animal models and in humans when studying BM progenitor hematopoiesis [170–173]. A subset of these myeloid cells termed myeloid-derived suppressor cells (MDSCs) are increased in aged individuals and are highly associated with cancer development and progression. For example, in a study of colorectal cancer patients, a positive correlation was observed for circulating MDSCs and overall tumor burden [174]. These cells suppress anti-tumor responses through mechanisms that differ from Treg cell immunosuppression, but nonetheless are correlated with age-related cancer incidence [175, 176]. MDSC induction has been attributed to pro-inflammatory cytokines, such as IL-6, which we know to be increased during inflammaging [175, 177]. Therefore, perhaps if the thymic niche was rejuvenated for enhanced lymphopoesis and the inflammatory environment during inflammaging was dampened, rebalance of myeloid-to-lymphoid hematopoiesis could reduce MDSC induction and alleviate their role in cancer progression.

Taken together, the axis connecting age-related thymic involution, T cell immunosenescence and chronic inflammatory environment, to tumorigenesis and tumor metastasis is intriguing, but the current knowledge is insufficient, and more evidence is necessary.

## Key Triggers Associated with Induction of Age-Related Thymic Involution

Age-related thymic involution is characterized by a reduction in thymic size and thymocyte numbers as well as overt remodeling of the thymic microstructure [70]. The thymus is a meshwork structure, in which thymocytes of hematopoietic origin undergo development and selection within various compartments containing TECs of non-hematopoietic origin [41, 91]. The aged, involuted thymus declines in both TECs and thymocytes. The initial question was which cellular compartment contained the primary defect that triggered thymic involution.

It has been noted that BM hematopoietic stem cells (HSCs) are decreased with age [34] and exhibit a skewed developmental pathway resulting in a decreased ratio of lymphoid-to-myeloid cells [170–172, 178]. Since the thymocyte progenitor cells immigrate to the thymus from the BM, this raised a natural question of whether aged BM-derived HSC lymphoid progenitors are sufficiently able to seed the thymus. Therefore, many studies have investigated this aspect. The outcome was that aged HSCs contain defects [34] that could contribute to insufficient thymic seeding by early T-cell progenitors (ETPs) [35], culminating in decreased thymic output with age [179]. The conclusion was largely based on BM transplantation experiments in mice [180] or *in vitro* fetal thymic organ culture experiments to assess ETP proliferation [35]. Therefore, aged HSCs and ETPs were regarded as having an intrinsic defect [181]. This conclusion was confirmed using BM aspirate samples from young and elderly patients in which gene expression profiling of the HSCs showed differential gene expression associated with skewed myeloid lineage determination, however, it is possible that circulating factors in the aged periphery, such as cytokines could be initiating such lineage shifts [170].

Importantly, the role of non-hematopoietic origin TECs and BM stromal cells in age-related thymic involution was neglected by these studies. We focused on the role of HSC/thymocyte niche cells by several experimental designs [1]:. For BM transplantation, we avoided the usual whole body irradiation and reduced artifacts of *in vitro* HSC manipulation [38, 182] by instead utilizing young or aged IL-7R knockout mice as recipients [38, 183, 184], since these mice have a BM niche that is accessible to seeding exogenous BM cells without irradiation [183, 185]. After BM cell engraftment, the young BM cells exhibited a young phenotype in young recipients, but the young BM cells exhibited an old phenotype in aged recipients [38]. This suggests that the microenvironmental cells, rather than the HSCs, directs BM cell aging [39] [2]. .We also performed transplantation of “microenvironmental niche”, i.e. fetal mouse thymi, into young or aged mice under the kidney capsule, in which BM progenitors from the host mice directly seed the engrafted fetal thymus *in vivo* [182]. After engraftment, BM progenitors from young and aged mice developed equally well in the young engrafted thymus [182]. These comprehensive experiments provide substantial evidence demonstrating that the aged non-hematopoietic microenvironment, rather than aged HSCs or ETPs [39], mediates age-related thymic involution [11]. The result can be explained by the “seed and soil” theory, which describes how stem niches (soil) direct progenitor cell (seed) fate [186–188], and how thymocytes and the stromal microenvironment (TECs) cross-talk in the thymus [40], leading us to conclude that age-related thymic involution begins with defects in the TEC compartment. Therefore, it is possible that diminished thymic factors, such as IL-7 [189], in the aged, involuted thymus could provide signals to HSCs that facilitates the shift in lymphoid-to-myeloid lineage observed in aged HSCs.

To identify which specific factors mediate cellular and molecular TEC aging, many groups have performed substantial work. They found many age-related TEC influencing factors, including sex steroids, cytokines, transcription factors, and microRNAs, but the single most predominant mechanistic factor currently accepted as causal to thymic involution is the TEC autonomous transcription factor *FOXN1*, which is uniquely expressed in epithelial cells of the thymus and skin to help regulate epithelial cell differentiation [190, 191]. It is required for thymic organogenesis and responsible for thymocyte development [42], as well as hair follicle development in the skin [192, 193]. Many past and current studies utilize nude mice as a model, which exhibit a null mutation in *FOXN1* resulting in the lack of hair and thymus, and therefore lack of T cells [194, 195].

*FOXN1* expression is reduced in the aged thymus and has even been described as one of the first markers of the onset of thymic involution [196, 197]. The question of the cause-and-effect relationship of *FOXN1* decline and thymic involution had been largely under debate until the advent of a *FOXN1* cKO mouse model [198]. In this model, the murine *FOXN1* gene is *loxP*-floxed and the ubiquitous Cre-recombinase with tamoxifen (TM)-inducible fused estrogen receptor blocker (uCreER^T^) is introduced through crossbreeding [199], in which a low level of spontaneous activation takes place over time, even without tamoxifen (TM) induction [200, 201]. This causes a gradual excision of the *FOXN1*^*flox/flox*^ gene over time and results in a progressive loss of *FOXN1* with age. The thymic involution that results is positively correlated with reduced *FOXN1* levels [202]. Furthermore, supplying exogenous FOXN1, such as via plasmid [202] or transgene [203, 204], into the aged thymus greatly reduces thymic atrophy and improves thymic function. Additionally, use of *FOXN1* reporter mice have enabled further elucidation of the timeline and kinetics of thymic atrophy with age [205]. It is now largely accepted that progressively decreased *FOXN1* expression resulting from age introduces defects in TEC homeostasis, resulting in age-related thymic involution.

## Trends for Rejuvenation of Age-Related Thymic Involution

Since the T cell compartment is implicated in so many aspects of inflammaging and immunosenescence, we believe that one potential strategy for ameliorating the effects of inflammaging is via rejuvenation of the aged, involuted thymus. By restoring thymic function, we would repair the defects in negative selection and rebalance tTreg generation. Currently, there are several strategies for rejuvenation of thymic involution in the literature, some of which target systemic T cell immunity and others focus on the thymus itself.

### *FOXN1*-TEC axis

Since the TEC-autonomous factor *FOXN1* is heavily implicated in onset and progression of age-related thymic involution, several strategies attempt to target the *FOXN1*-TEC axis to specifically restore TEC function.

#### Cellular therapy

First, some TEC stem cell-based strategies include utilization of human embryonic/pluripotent stem cells [206–208], *FOXN1*^eGFP/+^ knock-in epithelial cells [209], and young TEC-based [210] or inducible TEC-based [211] strategies. These all involve engraftment of exogenous *FOXN1* producing cells into thymic tissue. One such group directly transplanted TECs from newborn mice intrathymically into middle-aged recipients and observed renewed growth of the thymus as well as enhanced T cell generation [210].

Another group generated induced TECs (iTECs) from exogenous *FOXN1*-overexpressing mouse embryonic fibroblasts (MEF) cells by initiating the exogenous *FOXN1* expression that converted MEF cells into epithelial-like cells *in vitro* [211]. Engraftment of these iTECs under the kidney capsule of syngenic adult mice created a *de novo* ectopic thymus. Host T cell progenitors seeded the *de novo* thymus-like organ generated by the transplant and normal thymocyte distributions were observed after 4 weeks. Additionally, typical thymus microstructure was seen in the *de novo* thymic engraftment [211].

#### Cytokine therapy

There are also some cytokine-to-TEC based therapies, such as keratinocyte growth factor [212, 213] and IL-22 [214–216]. Many of these animal studies observed thymic regrowth and improved thymopoiesis, however, they largely used models of acute thymic insult, such as irradiation. As for chronic age-induced thymic atrophy, IL-22 may offer more benefits for improved thymic microenvironment since one study saw correlative up-regulation of IL-22 and FOXN1 after acute thymic insult in mice [217]. Though promising, the extent of crosstalk between IL-22 and FOXN1 within the thymus remains to be determined.

Another cytokine under investigation is IL-7, which is normally secreted by TECs, and helps mediate thymopoesis. IL-7 is reduced in the aged thymus [189] but its role in other aspects of immune system development and proliferation presents a challenge in approaching IL-7 supplementation as a systemic therapy. One such example is a study administering recombinant IL-7 to aged rhesus macaques, which demonstrated little effect of thymic function, but did result in enhanced peripheral T cell proliferation [218]. Several clinical studies have been conducted with systemic IL-7 treatment to boost peripheral T cell proliferation after chemotherapy or after infection or vaccination to amplify immune responses, but these were more focused on peripheral expansion (reviewed [219]). Importantly, peripheral T cell subsets express differing levels of the IL-7 receptor, effecting the extent of IL-7-induced expansion (i.e. more CD8^+^ T cells expand compared to CD4^+^ T cells with minimal expansion of Treg cells) [219].

However, IL-7 targeting to the aged thymus may restore more balanced T cell development in the elderly. For example, one study generated a plasmid-delivered IL-7 fusion protein that combined IL-7 with the N-terminal extracellular domain of CCR9 to target this protein to the thymus and reduce adverse systemic effects of increased IL-7 [220]. They observed restoration of thymic architecture and enhanced cellularity, similar to that of young animals, in the thymus of aged animals that received fusion protein treatment compared to unaltered IL-7 and control plasmid groups [220]. This study holds great promise as a targeted cytokine therapy.

Finally, since TCR repertoire contraction is a contributor to immune insufficiency in aging, it is interesting to note that systemic treatment with recombinant IL-7 resulted in increased TCR diversity in patients who had undergone bone marrow transplant [221]. Again, given the other effects of systemic IL-7, this may not present a realistic therapy for thymic atrophy alone, but it does compel further study into how some of these cytokines and circulating factors may impact T cell development and selection independently and/or synergistically with age-related thymic involution.

#### Gene therapy

Similar to the TEC-based cellular therapy, some groups have utilized genetically-based methods to enhance exogenous *FOXN1* expression, either with *FOXN1* cDNA plasmid or *FOXN1* transgenes) [202–204]. One group intrathymically injected plasmid vectors carrying *FOXN1*-cDNA into middle-aged and aged mice and observed partial rescue of thymic size and thymocyte numbers compared to empty vector controls [202]. Another group, utilizing an inducible *FOXN1* overexpression reporter gene system, showed *in vivo* upregulation of *FOXN1* expression in middle-aged and aged mice resulted in increased thymic size and thymocyte numbers [204]. They also observed enhanced ETP cell numbers, and the mTECs:cTECs ratio was restored to normal levels [204]. Moreover, these targeted *FOXN1* gene therapies also show great promise for rejuvenation of aged thymic structure and function.

### Periphery – thymus axis

#### Growth hormones

Decline in growth hormone during aging has been suggested to contribute to age-related thymic involution and animal studies using growth hormone supplementation show rescue of thymic atrophy, increased T cell progenitor recruitment into the thymus, as well as enhanced thymic microenvironmental cytokine production [222–224]. Studies of growth hormone date back to the early 1999s after the observations that TECs express growth hormone receptors and that insulin-like growth factor is expressed in the thymus [225–227]. Studies of insulin-like growth factor 1 (IGF-1), which is closely related to growth hormone, show similar thymic functional and structural improvements upon increased IGF-1 levels in aged mice [222, 228]. Although, the effects of crosstalk between growth hormones and many other neuroendocrine hormones with thymocytes and TECs are under investigation, these systemic pathways are extremely interwoven and thus difficult to compartmentally delineate [222, 228].

#### Sex hormones

The effects of sex hormones on the thymus have long been characterized, with the earliest reports of thymic atrophy correlating with adolescence and reproductive hormones dating back to a 1904 study in cattle [229]. Early studies using castration and sex steroid antagonists in both male mice and male patients receiving androgen blockade for prostate cancer therapy demonstrated phenotypes varying from delayed onset of thymic involution to complete thymic regeneration [230–233]. Most of these early studies, however, focused primarily on phenotypic data, such as an increase of thymopoiesis, with insufficient mechanistic results. Generally, the rejuvenation is thought to occur in the TEC compartment because androgen receptors are expressed by TECs [234]. One of the potential mechanisms reported was that sex steroids inhibit cTEC expression of Notch ligand Delta-like 4 (DLL4), shown in one study utilizing a luteinizing hormone-releasing hormone blockade that saw enhanced thymopoesis after blockade in mice [235]. DLL4 is an important factor for promoting T cell differentiation and development. It remains unclear whether Notch ligands (there are four types) are decreased in the aged thymus and how this might play a role in decreased thymopoiesis with age.

In contrast, other studies of thymic rejuvenation through sex steroid ablation exhibited in the least, only a short-lived rejuvenation, and at most no influence whatsoever on thymic involution in mice [236]. Others suggest that the observable thymic restoration can be transient (only 2 weeks) but harmful, asserting that the “rejuvenated” thymus potentially produces more harmful T cells and increasing self-reactivity [237]. In support of the opinion that sex hormone ablation may cause detrimental autoimmune implications, a human study, which used medical castration resulted in a declined % CD4^+^CD25^+^ Treg cells and increased NK cells, which may compromise immune tolerance [238].

Recently, studies on sex hormones and their impact on thymocyte selection of the TCR repertoire via *AIRE* gene expression by TECs in the thymus demonstrate that there are differences in males and females in both mouse and human samples [239–241]. Androgens from males promote *AIRE* expression in mTECs to enhance thymocyte negative selection, while estrogens reduce *AIRE* expression, dampening thymocyte negative selection and potentially increasing autoimmunity [240, 241]. Therefore, these hormones may mediate thymic functionality to a greater extent than simply structural atrophy. In light of this, sex steroid antagonists or castration-based rejuvenation of thymic aging may have more disadvantages (inducing autoimmune predisposition in the elderly) than advantages.

#### Blood-borne factors

Of note, there are likely circulating factors that impact age-related thymic involution, including proteins, mRNAs, microRNAs and other signaling molecules. One method to test this is a heterochronic parabiosis model, in which young and aged mice are surgically conjoined resulting in mutual influence of blood-borne factors. These experiments, however, have not demonstrated rejuvenation of the aged thymus [242–250]. Conversely, when serum-derived extracellular vesicles, which carry cellular factors throughout the body, were taken from young mice and given to aged hosts, partial thymic rejuvenation with increased negative selection signaling was observed [251]. Interestingly, we also observed decreased levels of circulating pro-inflammatory IL-6, suggesting rescue from inflammaging following treatment with these young serum-derived extracellular vesicles [251]. Further work to elucidate the mechanism of ameliorated inflammaging phenotype is necessary, as it could be due to increased targeted deletion of senescent cells in the periphery causing less SASP secretion, enhanced Treg production, or other unknown mechanisms.

#### Life-Style/Physical Exercise

Finally, life-style habits should not be overlooked pertaining to immune health and healthy aging. Indeed, CT scans of patient thymus tissue demonstrate that advanced fatty degeneration of the thymus is positively correlated with increased BMI and with smoking [252]. Additionally, physical exercise has demonstrated countless benefits for immune health, some of which have recently been reported. One such study has documented an intriguing correlation between physical exercise and improved thymic function in elderly patients. This in-depth study compared numerous aspects of immunosenescence and thymic output in aged adults who participated in high levels of regular exercise for much of their adult lives and aged adults who had been inactive [253]. This study found that the aged individuals who maintained physical exercise regimens exhibited reduction in typical decline in thymic output, decreased markers of inflammaging, such as reduced serum IL-6, and increased serum IL-7 and IL-15, which may foster thymic health and function [253]. The age-associated increase in Th17 phenotype was also significantly lessened in the aged cohort with physical exercise and lower peripheral Treg cell numbers were observed in these individuals compared to the inactive aged cohort [253]. Though not all aspects of immunosenescence were lessened in the exercising cohort, as both groups maintained the age-related accumulation of senescent T cells, this study does present some compelling findings. This group published a recent review and discussed the direct cross-talk between skeletal muscles during exercise and the immune compartment, even describing exercise as a potential adjuvant to immunizations, as some studies have also shown enhanced T cell priming and increased naïve T cell frequency [254]. Therefore, it is significant to mention the effects of physical exercise and overall healthy life-style habits on immune health and directly on thymic health over the lifespan.

In sum, there are many varied avenues for restoration of aged thymic structure and function as well as its influences on inflammaging. Many of these rejuvenation strategies focus on the TEC compartment, since decline in TECs and TEC-associated factors are implicated in thymic involution onset and progression, however, the role of other systemic players are still under investigation. Additionally, each strategy has disadvantages. For example, intrathymic injection of newborn TECs can rejuvenate middle-aged thymus [210], but the source of newborn TECs is limited and may not be ideal as a translational therapy. Additionally, generation of an ectopic *de novo* thymus under the kidney capsule [211] can generate naïve T cells, but this does not remedy the increased self-reactive T cells released by the original atrophied thymus remaining in the host. Also, the use of thymus-targeted cytokines may be beneficial, but caution is needed, as systemic cytokine therapies usually encompass adverse effects. Moreover, continued investigation is required for future development of practical and effective interventions for age-related thymic involution and inflammaging.

## Conclusion

Age-related thymic involution is a dynamic process that impacts overall T cell development and central T cell tolerance establishment throughout life. Immunosenscence and inflammaging describe two opposing arms of the aged immune system: immune insufficiency, with regard to infection, vaccination, and tumor surveillance, coupled with increased self-reactivity and chronic, systemic inflammation. The contributions of the aged thymus to the manifestations of immunosenscence and inflammaging have recently come to be appreciated. However, continued investigation into their synergy in the aged immune system is needed. Additionally, as we shift our focus towards improving quality of life with age, research into potential avenues for reversing the adverse effects of age-related thymic involution on the aged T cell immune system is of paramount importance. Moreover, there are numerous areas still to explore in this field with far-reaching applications.

## Data Availability

Not applicable.

## Abbreviations

Aβ: Amyloid beta
AD: Alzheimer’s disease
AIRE: Autoimmune regulator
BBB: Blood-brain barriers
BM: Bone marrow
cKO: conditional knock-out
CNS: Central nervous system
CP: Choroid plexus
CTL: Cytotoxic T lymphocyte
ETP: Early T-cell progenitor
FOXN1: Forkhead box N1
MEF: Mouse embryonic fibroblasts
MDSC: Myeloid-derived suppressor cell
MHC-II: Major histocompatibility complex class II
RTE: Recent thymic emigrant
SASP: Senescence-associated secretory phenotype
SSC: Senescent somatic cell
TCR: T cell antigen receptor
TEC: Thymic epithelial cell (mTEC: medullary TEC cTEC: cortical TEC)
TF: Transcription factor
Treg cell: Regulatory T cell (tTreg cell: thymic Treg cell pTreg cell: peripheral Treg cell)
